# The prognostic value of combined TGF-β1 and ELF in hepatocellular carcinoma

**DOI:** 10.1186/s12885-015-1127-y

**Published:** 2015-03-11

**Authors:** Fei Ji, Shun-Jun Fu, Shun-Li Shen, Long-Juan Zhang, Qing-Hua Cao, Shao-Qiang Li, Bao-Gang Peng, Li-Jian Liang, Yun-Peng Hua

**Affiliations:** 1 Organ Transplant Center, the First Affiliated Hospital, Sun Yat-sen University, Guangzhou, 510080 P. R. China; 2 Department of Hepatopancreaticobiliary Surgery, The Second Affiliated Hospital of Guangzhou University of Chinese Medicine (Guangdong Provincial Hospital of TCM), Guangdong Provincial Hospital of Traditional Chinese Medicine, Guangzhou, 510120 P. R. China; 3 Department of Liver Surgery, the First Affiliated Hospital, Sun Yat-sen University, Guangzhou, 510080 P. R. China; 4 Laboratory of Surgery, the First Affiliated Hospital, Sun Yat-sen University, Guangzhou, 510080 P. R. China; 5 Department of Pathology, the First Affiliated Hospital, Sun Yat-sen University, Guangzhou, 510080 P. R. China

**Keywords:** Transforming growth factor, Embryonic liver fodrin, Hepatocellular carcinoma, Prognosis, Biomarkers

## Abstract

**Background:**

Tumor suppression of Transforming Growth Factor (TGF-β) signaling pathway requires an adaptor protein, Embryonic Liver Fodrin (ELF). Disruption of ELF expression resulted in miscolocalization of Smad3 and Smad4, then disruption of TGF-β signaling. However, the prognostic significance of ELF for hepatocellular carcinoma (HCC) hasn’t been clarified. This study aimed to investigate whether measuring both TGF-β1 and ELF provides a more powerful predictor for HCC prognosis than either marker alone.

**Methods:**

TGF-β1 and ELF protein were detected by immunohistochemistry. The relationship between TGF-β1/ELF expression and patients’ clinicopathologic factors was analyzed. The association between TGF-β1/ELF expression and disease-free survival and overall survival was analyzed by Kaplan-Meier curves, the log-rank test, and Multivariate Cox regression analyses.

**Results:**

The expression of TGF-β1 in HCC tissues was significantly higher than that in normal liver tissues. Conversely, the expression of ELF in HCC tissues declined markedly. ELF protein was correlated with HBsAg, tumor size, tumor number, TNM and recurrence. Data also indicated a significant negative correlation between ELF and TGF-β1. Patients with high TGF-β1 expression or/and low ELF expression appeared to have a poor postoperative disease-free survival and overall survival compared with those with low TGF-β1 expression or/and high ELF expression. Furthermore, the predictive range of ELF combined with TGF-β1 was more sensitive than that of either one alone.

**Conclusions:**

TGF-β1 and ELF protein are potential and reliable biomarkers for predicting prognosis in HCC patients after hepatic resection. Our current study has demonstrated that the prognostic accuracy of testing can be enhanced by their combination.

## Background

Hepatocellular cancer (HCC) is one of the most common, aggressive malignancies, the third leading cause of cancer-related deaths worldwide (World Health Organization Report, 2006) [1-3]. Although surgical resection, percutaneous ablation and liver transplantation are considered as the curative treatments for HCC, the long-term prognosis of patients undergoing potentially curative treatments is still poor. Fully 60% to 70% of patients develop recurrence or metastasis within 5 years after resection [4,5]. It is therefore a very important and urgent task to find an effective biomarker to identify patients with a high risk of recurrence or metastases, and provide personalized therapy according to the predicted risk of recurrence.

The transforming growth factor β (TGF-β) signaling pathway is known to play an important role in multiple cellular processes, including cell growth, differentiation, adhesion, migration, apoptosis, extracellular matrix formation and immunosuppressant [6-9]. TGF-β signals are conveyed from type I and type II transmembrane serine/threonine kinase receptors to the intracellular mediators-Smad2 and Smad3, which further complex with Smad4, translocate to the nucleus and bind to Smad-binding elements (SBE) in target gene promoters, thereby activating its targets, such as p21, p15, p16, p27 [10-14]. TGF-β is particularly active as a profound tumor suppressor by prohibiting cell cycle progression and arresting cells in early G1 phase. However, misregulation of TGF-β signaling promotes tumor growth and invasion, evasion of immune surveillance, and cancer cell dissemination and metastasis [11-14]. In HCC tissues, the overexpression of TGF-β1 was found and correlated with carcinogenesis, progression, and prognosis of HCC, while normal hepatocytes had not any TGF-β1 staining [15]. In our previous study, we found hepatocarcinogenesis could be closely related to the low expression of Smad4 and phosphorylated Smad2, and the high expression of TGF-β1 and Smad7 in advanced stage of liver cirrhosis [16].

Embryonic Liver Fodrin (ELF), also named as β2-spectrin (β2SP), first isolated from foregut endodermal stem cell libraries, functions as a Smad3/4 adaptor protein, plays critical roles in the proper control of Smad access to activating receptors involved in regulation of TGF-β signaling [17-19]. Interestingly, ELF is a key suppressor of tumorigenesis [20,21]. Disruption of ELF expression by gene knockout was found to result in miscolocalization of Smad3 and Smad4, and disruption of TGF-β signaling [22]. About half of mice with heterozygous deletion of ELF developed hepatocellular carcinoma, and 90% of ELF^+/−^/Smad^4+/−^ mice developed gastric cancer and other gastrointestinal cancers [23,24]. Loss of ELF may play a role in the malignant transformation of hepatic progenitor/stem cells [22]. However, the prognostic value of ELF for HCC is not well-known. Testing the combination of TGF-β1 and ELF as a predictor for HCC prognosis is also merits study.

In the present study, we examined the pattern of expression of TGF-β1 and ELF in HCC tumor tissues and normal tissues. Together with the known function, it is therefore of interest to investigate that TGF-β1 and ELF protein are potential and reliable biomarker for predicting prognosis in HCC patients after hepatic resection, and prognostic accuracy of testing can be enhanced by their combination in the patients with HCC.

## Methods

### Patients and tissue samples

A total of 84 adult patients with HCC who underwent hepatic resection in the Department of Hepatobiliary Surgery, First Affiliated Hospital of Sun Yat-sen University between June 2007 and October 2009, were enrolled in this study, including 68 males and 16 females with an average age of 48 years (range 23 to 75 years). Written informed consent was obtained from all patients, and the study was conducted in accordance with the protocol approved by the Declaration of Helsinki and the guidelines of the Ethics Review Committee of First Affiliated Hospital of Sun Yat-sen University. In addition, normal liver tissues were collected from patients with cavernous hemangioma of liver or patients with intrahepatic stones.

The diagnosis of HCC met the criteria of the American Association for the study of Liver Disease [25]. The volume of liver resection and the surgical procedures were decided by tumor size, tumor location, and liver functional reserve based on a multidisciplinary team meeting every week. Tumor stages were classified according to the tumor-node-metastasis (TNM) system of the International Union Against Cancer by the American Joint Committee [26]. The histologic grade of tumor was assigned according to the Edmondson Steiner grading system [27]. Fresh HCC tissues and HCC adjacent tissues were collected within 30 minutes after resection. These tissues were fixed with 10% formalin and then embedded in paraffin.

### Immunohistochemical analysis

The techniques have been described previously [16]. The sections were incubated with pre-diluted primary Rabbit polyclonal anti-ELF antibody (ab72239, Abcam, USA) at a dilution of 1:100, with Rabbit monoclonal anti-TGF-β1 antibody (Y369, Bioworld, USA) at dilution of 1:100, at 4°C overnight. Negative controls were treated the same way, omitting the primary antibodies.

### Evaluation of immunohistochemical staining

The immunohistochemical staining in the tissue was scored independently by 2 pathologists blinded to the clinical data, by applying a semiquantitative immunoreactivity score (IRS) reported elsewhere [28-30]. Category A documented the intensity of immunostaining as 0–3 (0, negative; 1, weak; 2, moderate; 3, strong). Category B documented the percentage of immunoreactive cells as 0 (less than 5%),1 (6%–25%), 2 (26%–50%), 3 (51%–75%), and 4 (76%–100%). Multiplication of category A and B resulted in an IRS ranging from 0 to 12 for each tumor or nontumor. Sections with a total score of 0 or 1 or 2 were defined as negative (−), score of 3 or 4 were defined as weakly positive (+), score of 6 or 8 were defined as moderately positive (++), score of 9 or 12 were defined as strongly positive (+++). For categorical analyses, the immunoreactivity was graded as low level (total score < =4) or high level (total score >4).

### Follow-up

The postoperative patients were followed up once a month during the first half year post-operatively and every 3 months thereafter. Serum AFP level and abdominal ultrasonography were done routinely during the postoperative review. Computed tomography (CT) was performed every 3 to 6 months together with chest radiographic examination. The endpoint of study was December 2013. Survival time was calculated from the date of surgery to the date of death or to the last follow-up. Date of death was obtained from patient records or patients’ families through follow-up telephone calls. Date of death for each case was double verified by local civil affairs department and public security department. The median follow-up period was 39 months (range 3 to 81 months).

Recurrence or metastasis was detected by imaging examination such as ultrasonography, contrast-enhanced ultrasonography, CT, magnetic resonance imaging (MRI), hepatic arterial angiography, or positron emission tomography -CT (PET-CT). Isolated increases in serum AFP were not regarded as recurrent events. Once tumor recurrence was verified, patients received the appropriate further treatments, including repeat liver resection, radiofrequency ablation, percutaneous ethanol injection, chemoembolization, and/or molecular targeting therapy by sorafenib.

### Statistical analysis

Statistical analyses were carried out using the SPSS v 13. 0 software (Chicago, IL, USA). The Wilcoxon W rank sum test and chi-square test was used to compare qualitative variables. Spearman correlation was used to investigate the correlation between ELF and TGF-β1 expression. Survival curves were calculated using the Kaplan-Meier method and were compared by a log-rank test, illustrated by survival plots. The Cox proportional hazards model was used to determine the independent risk factors associated with prognosis. *P* < 0.05 was considered statistically significant.

## Results

### The low expression of ELF and the high expression of TGF-β1 in HCC tissues

**Figure 1:**
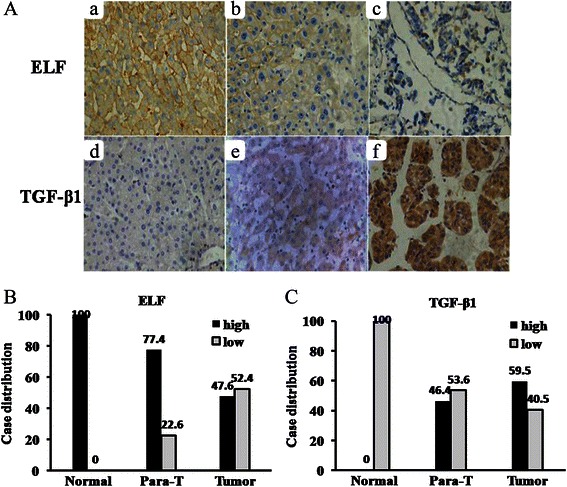
**Expression of ELF and TGF-β1 protein. (A)** Immunohistochemical staining in different tissues is shown. Normal liver tissues (Aa and Ad), HCC adjacent tissues (Ab and Ae), HCC tissues (Ac and Af) (original magnification × 400). **(B)** and **(C)** Case distribution of ELF/TGF-β1 expression in normal liver tissues (Normal), HCC adjacent tissues (Para-T) and HCC tissue (Tumor).

Using immunohistochemical staining, we examine expression of ELF and TGF-β1 on 20 normal liver tissues, 84 HCC samples and adjacent tissues. All normal liver tissues expressed high level of ELF (20/20). In HCC adjacent tissues, there was a 77.4% high expression rate for ELF (65/84). However, the ELF high expression rate declined to 47.6% (40/84) in HCC tissues. There was significant difference among the groups examined (*P* < 0.001) (Table 1, Figure 1A, B). On the contrary, the expression rate of TGF-β1 in HCC tissues (59.5%, 50/84) was significantly higher than that in the normal liver tissues (0, 0/20, *P* < 0.001), but not in HCC adjacent tissues (46.4%, 39/84, *P* = 0.089, Table 2, Figure 1A, C). These results suggested that there was the low expression of ELF and high expression of TGF-β1 in HCC tissues.

**Table 1:** **The expression of ELF in HCC**

Group	n	Expression of ELF	
		High	Low
Normal liver tissues	20	20(100.0%)	0(0.0%)
Adjacent tissues*	84	65(77.4%)	19(22.6%)
HCC tissues*^#^	84	40(47.6%)	44(52.4%)

*compared with Normal liver tissues, *P* < 0.001 (by chi-square test).

^#^compared with Adjacent tissues, *P* < 0.001 (by chi-square test).

**Table 2:** **The expression of TGF-β1 in HCC**

Group	n	Expression of TGF-β1	
		High	Low
Normal liver tissues	20	0(0.0%)	20(100.0%)
Adjacent tissues*	84	39(46.4%)	45(53.6%)
HCC tissues*	84	50(59.5%)	34(40.5%)

*compared with Normal liver tissues, *P* < 0.001 (by chi-square test).

### Correlation between TGF-β1/ELF expression and 16 clinico-pathologic characteristics in HCC

In order to further understand the prognostic value of TGF-β1/ELF expression for HCC after resection, the relationships between the expression of these proteins and 16 clinico-pathologic characteristics, such as age, gender, HCC family, HBsAg, ALT, AFP, cirrhosis, ascites, PVTT, tumor size, tumor number, tumor differentiation, tumor encapsulation, TNM stage, recurrence and complication, were analyzed. The expression level of ELF was negatively correlated with HBsAg (*P* =0.04), tumor size (*P* = 0.010), tumor number (*P* = 0.001), TNM stage (*P* = 0.027) and recurrence (*P* < 0.001). As predicted, TGF-β1 expression was positively associated with the tumor size (*P* = 0.001), tumor number (*P* = 0.003), TNM stage (*P* = 0.002) and recurrence (*P* < 0.001), too (Table 3). In addition, we found the significant negative correlation between ELF and TGF-β1 expression patterns by using Spearman correlation (r = −0.271, *P* = 0.013, Table 4).

**Table 3:** **Correlation between the clinicopathological characteristics and expression of ELF and TGF-β1 in the 84 HCC patients**

*Variables*	*Cases*	*ELF expression*		*P value*	*TGF-β1 expression*		*P value*
		Low	High		Low	High	
Age(yrs)							
> = 60	16	7(43.8%)	9(56.2%)	0.442	9(54.4%)	7(54.4%)	0.325
<60	68	37(54.4%)	31(45.6%)		29(42.6%)	39(57.4%)	
Sex							
Male	68	37(54.4%)	31(45.6%)	0.44	27(39.7%)	41(60.3%)	0.77
Female	16	7(43.8%)	9(56.2%)		7(43.8%)	9(56.2%)	
HCC family history							
Yes	6	2(33.3%)	4(66.7%)	0.83	3(50.0%)	3(50.0%)	0.95
No	78	42(53.8%)	36(46.2%)		31(39.7%)	47(60.3%)	
HbsAg							
Positive	72	41(56.9%)	31(43.1%)	0.04	29(40.3%)	43(59.7%)	0.93
Negative	12	3(25.0%)	9(75.0%)		5(41.7%)	7(58.3%)	
ALT(U/L)							
≥80	9	3(33.3%)	6(66.7%)	0.39	1(11.1%)	8(88.9%)	0.12
<80	75	41(54.7%)	34(45.3%)		33(44.0%)	42(56.0%)	
PLT(×10^9^)							
>100	74	41(55.4%)	33(44.6%)	0.24	28(37.8%)	46(62.2%)	0.32
≤100	10	3(30.0%)	7(70.0%)		6(60.0%)	4(40.0%)	
Cirrhosis							
Yes	64	32(50.0%)	32(50.0%)	0.43	27(42.2%)	37(57.8%)	0.57
No	20	12(60.0%)	8(40.0%)		7(35.0%)	13(65.0%)	
AFP(ug/L)							
≥20	48	28(58.3%)	20(41.7%)	0.21	19(39.6%)	29(60.4%)	0.85
<20	36	16(44.4%)	20(55.6%)		15(41.7%)	21(58.3%)	
Tumor size (cm)							
≥5	50	32(64.0%)	18(36.0%)	0.01	13(26.0%)	37(74.0%)	0.001
<5	34	12(35.3%)	22(64.7%)		21(61.8%)	13(38.2%)	
Tumor number							
Single	62	26(41.9%)	36(58.1%)	0.001	31(50.0%)	31(50.0%)	0.003
Multiple	22	18(81.8%)	4(18.2%)		3(13.6%)	19(86.4%)	
Differentiation							
I-II	62	31(50.0%)	31(50.0%)	0.46	27(43.5%)	35(56.5%)	0.34
III-IV	22	13(59.1%)	9(40.9%)		7(31.8%)	15(68.2%)	
TNM stage							
I-II	55	24(43.6%)	31(56.4%)	0.03	29(52.7%)	26(47.3%)	0.002
III-IV	29	20(69.0%)	9(31.0%)		5(17.2%)	24(82.8%)	
PVTT							
Yes	11	8(72.7%)	3(27.3%)	0.15	2(18.2%)	9(81.8%)	0.11
No	73	36(49.3%)	37(50.7%)		32(43.8%)	41(56.2%)	
Tumor encapsulation							
Complete	64	30(46.9%)	34(53.1%)	0.07	29(45.3%)	35(54.7%)	0.11
None	20	14(70.0%)	6(30.0%)		5(25.0%)	15(75.0%)	
Recurrence							
Yes	56	39(69.6%)	17(30.4%)	<0.001	12(21.4%)	44(78.6%)	<0.001
No	28	5(17.9%)	23(82.1%)		22(78.6%)	6(21.4%)	
Complication							
No	73	41(56.2%)	32(43.8%)	0.07	31(42.5%)	42(57.5%)	0.34
Yes	11	3(27.3%)	8(72.7%)		3(27.3%)	8(72.7%)	

AFP, Alpha-fetoprotein; HBsAg, hepatitis B surface antigen; PLT, platelet; PVTT, portal vein tumor thrombi.

**Table 4:** **The correlationship between ELF and TGF-β1 in HCC**

*ELF*	*TGF-β1*			*r*	*P value*
	+++	++	- ~ +		
+++	7	4	12	−0.271	0.013
++	4	1	12		
**- ~ +**	20	14	10		

### Independent prognostic factors of HCC

To further identify the risk factors linked to postoperative Disease Free Survival (DFS) and Overall Survival (OS), ELF, TGF-β1 and 16 clinicopathologic factors were evaluated by univariate analysis and the Cox regression model. The univariate analysis showed that the significant prognostic factors for DFS of HCC were tumor number, portal vein tumor thrombus (PVTT), tumor encapsulation, TNM stage, ELF expression, and TGF-β1 expression. Similarly, the analysis showed that the significant factors for OS of HCC were tumor number, PVTT, tumor size, resection margin, tumor differentiation, TNM stage, ELF expression, and TGF-β1 expression (all *P* < 0.05). Using the Cox regression multivariate analysis, we found that PVTT, ELF expression, and TGF-β1 expression were the significant independent related factors for DFS (all *P* < 0.05), in addition, tumor differentiation (*P* = 0.029), PVTT (*P* = 0.011), ELF expression (*P* = 0.042) and TGF-β1 expression (*P* < 0.001) were the significant independent related factors for OS (Tables 5 and 6).

**Table 5:** **Prognostic factors for DFS and OS by univariate analysis**

Variables	*n*	DFS			*P*	OS			P
		1-yr	3-yrs	5-yrs		1-yr	3-yrs	5-yrs	
Sex									
Male	68	54.4%	36.8%	30.7%	0.53	79.4%	50.0%	41.2%	0.48
Female	16	56.3%	37.5%	37.5%		87.5%	56.3%	50.0%	
Age(yrs)									
<60	68	45.6%	32.4%	29.3%	0.15	82.4%	48.5%	39.7%	0.39
≥60	16	62.5%	56.3%	42.2%		75.0%	62.5%	56.3%	
HCC family history									
Yes	6	50.0%	33.3%	16.7%	0.57	83.3%	50.0%	50.0%	0.63
No	78	48.7%	37.2%	33.3%		79.5%	51.3%	42.3%	
PLT(×10^9^)									
<100	10	80.0%	60.0%	60.0%	0.07	100.0%	80.0%	70.0%	0.08
≥100	74	44.6%	33.8%	28.2%		78.4%	47.3%	39.2%	
HBsAg									
Positive	72	47.2%	38.9%	33.3%	0.90	79.2%	50.0%	44.4%	0.88
Negative	12	58.3%	25.0%	25.0%		91.7%	58.3%	33.3%	
AFP(μg/L)									
<20	36	52.8%	38.9%	38.9%	0.34	83.3%	50.0%	44.4%	0.75
≥20	48	45.8%	35.4%	26.7%		79.2%	52.1%	41.7%	
Ascites									
No	68	52.9%	39.7%	34.1%	0.14	83.8%	51.5%	44.1%	0.55
Yes	16	31.3%	25.0%	25.0%		68.8%	50.0%	37.5%	
Cirrhosis									
No	24	45.0%	35.0%	30.0%	0.78	95.0%	60.0%	45.0%	0.49
Yes	60	50.0%	37.5%	32.9%		76.6%	48.4%	42.2%	
Tumor number									
Single	62	59.7%	43.5%	36.9%	0.003	85.5%	61.3%	51.6%	<0.001
Multiple	22	18.2%	18.2%	18.2%		68.2%	22.7%	18.2%	
PVTT									
No	73	54.8%	41.1%	35.4%	<0.001	87.7%	56.2%	47.9%	<0.001
Yes	11	9.1%	9.1%	9.1%		36.4%	18.2%	9.1%	
Tumor size (cm)									
<5	34	64.7%	47.1%	39.2%	0.05	97.1%	67.6%	52.9%	0.04
≥5	50	38.0%	30.0%	28.0%		70.0%	40.0%	36.0%	
Tumor encapsulation									
None	20	30.0%	25.0%	15.0%	0.01	60.0%	45.0%	30.0%	0.08
Complete	64	54.7%	40.6%	37.7%		87.5%	53.1%	46.9%	
Resection margin									
<2 cm	45	40.0%	26.7%	24.4%	0.07	80.0%	40.0%	28.9%	0.01
≥2 cm	39	59.0%	48.7%	39.6%		82.1%	64.1%	59.0%	
Complication									
No	73	49.3%	38.4%	33.1%	0.37	84.9%	53.4%	45.2%	0.10
Yes	11	45.5%	27.3%	27.3%		54.5%	36.4%	27.3%	
Tumor differetiation									
I-II	62	54.8%	41.9%	35.1%	0.16	85.5%	56.5%	48.4%	0.04
III-IV	22	31.8%	22.7%	22.7%		68.2%	36.4%	27.3%	
TNM stage									
I-II	55	60.0%	43.6%	38.0%	0.01	90.9%	60%	52.7%	0.001
III-IV	29	27.6%	24.1%	20.7%		62.1%	34.5%	24.1%	
ELF expression									
Low	44	25.0%	15.9%	10.2%	<0.001	72.7%	31.8%	23.7%	<0.001
High	40	75.0%	60.0%	57.5%		90.0%	72.5%	65.0%	
TGFβ1 expression									
Low	34	79.4%	73.5%	62.0%	<0.001	94.1%	85.3%	76.5%	<0.001
High	50	28.0%	12.0%	12.0%		72.0%	28.0%	20.0%	

AFP, Alpha-fetoprotein; HBsAg, hepatitis B surface antigen; PLT, platelet; PVTT, portal vein tumor thrombi.

**Table 6:** **Prognostic factors for disease-free and overall survival by the multivariate Cox proportional hazards regression model**

*Variables*	*DFS*			*OS*		
	HR	95% CI	*P*	HR	95% CI	*P*
Tumor differentiation				0.498	0.266-0.932	0.029
PVTT	0.405	0.199-0.824	0.013	0.398	0.195-0.812	0.011
ELF expression	2.135	1.115-4.088	0.022	1.989	1.024-3.862	0.042
TGFβ1 expression	0.219	0.099-0.486	<0.001	0.210	0.093-0.474	<0.001

HR, hazard ratio; CI, confidence interval; PVTT, portal vein tumor thrombi.

### Low expression of ELF and high expression of TGF-β1 predict HCC patients’ poor prognosis

**Figure 2:**
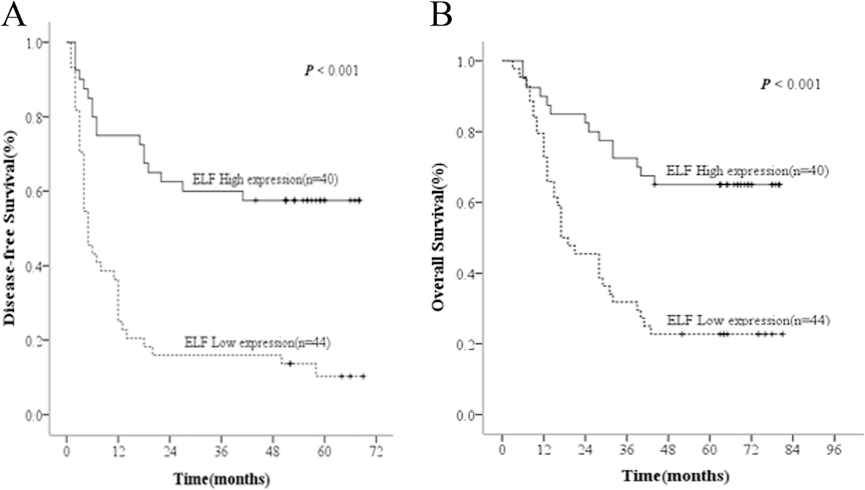
**Kaplan-Meier curves are shown for time to disease recurrence (A) and overall survival (B) among patients with high or low intratumoral ELF expression.**

Firstly, we divided 84 patients with HCC into 2 groups according to their ELF expression profiles: the low-expression group (n = 44) and the high-expression group (n = 40). Using the Kaplan-Meier method to analyze patients’ survival, we found that the 1-, 3- and 5-year DFS rates of the high-expression ELF group were remarkably higher than the low-expression group (75.0%, 60.0% and 57.5% vs 25.0%, 15.9% and 10.2%, respectively, *P* < 0.001) (Figure 2A), while the 1-, 3- and 5-year OS rates of the high-expression ELF group were significantly higher than those of the low-expression group (90.0%, 72.5% and 65.0% vs 72.7%, 31.8% and 23.7%, respectively, *P* < 0.001) (Figure 2B). Our findings therefore indicated that ELF expression levels were positively correlated with patients’ DFS and OS.

**Figure 3:**
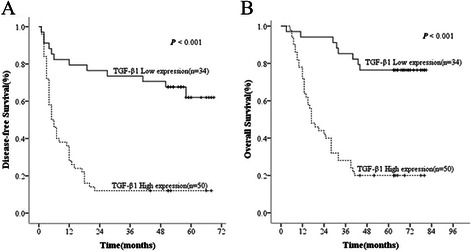
**Kaplan-Meier curves are shown for time to disease recurrence (A) and overall survival (B) among patients with high or low intratumoral TGF-β1 expression.**

Similarly, Two groups were divided from 84 HCC patients according to their TGF-β1 expression profiles: the low-expression group (n = 34) and the high-expression group (n = 50). We observed that the 1-, 3- and 5-year DFS rates of the low-expression TGF-β1 group were markedly higher than the high-expression group (79.4%, 73.5% and 62.0% vs 28.0%, 12.0% and 12.0%, respectively, *P* < 0.001) (Figure 3A). Also, the 1-, 3- and 5-year OS rates of the low-expression TGF-β1 group were significantly higher than those of the high-expression group (94.1%, 85.3% and 76.5% vs 72.0%, 28.0% and 20.0%, respectively, *P* < 0.001) (Figure 3B). These data suggested that TGF-β1 expression levels were negatively correlated with patients’ DFS and OS.

### The combination of TGF-β1 and ELF exhibits the improved prognostic accuracy for HCC

To analyze the prognostic value of combining TGF-β1 and ELF levels for HCC, we divided patients into the following four groups, such as: TGF-β1 high expression- ELF high expression group, TGF-β1 low expression- ELF high expression group, TGF-β1 high expression - ELF low expression group, TGF-β1 low expression- ELF low expression group. The data showed that the TGF-β1 low expression- ELF high expression group had the best DFS and OS rates, TGF-β1 low expression- ELF low expression group was the second best, the next was TGF-β1 high expression- ELF high expression group, whereas TGF-β1 high expression- ELF low expression group had the worst prognosis.

**Figure 4:**
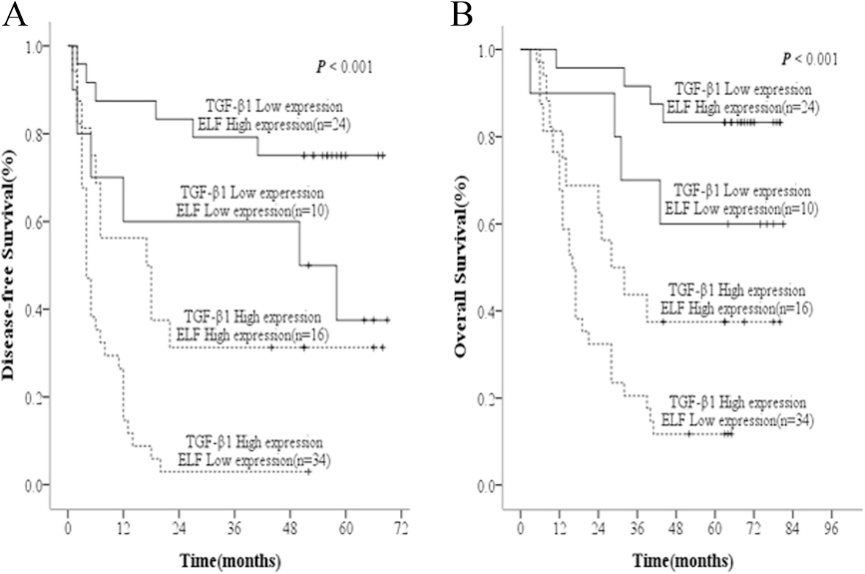
**The combination of ELF and TGF-β1 was found to enhance prognostic accuracy for HCC.** Disease-free survival curves **(A)** and overall survival curves **(B)**.

The 1-, 3- and 5-year DFS rates of TGF-β1 low expression- ELF high expression group (87.5%, 79.2% and 75.0%) were significantly higher than those of TGF-β1 high expression- ELF high expression group (56.3%, 31.3% and 31.3%, *P* = 0.003) and TGF-β1 high expression- ELF low expression group (26.5%, 2.9% and 2.9%, *P* < 0.001). The 1-, 3- and 5-year OS rates of TGF-β1 low expression- ELF high expression group (95.8%, 91.7% and 83.3%) were also significantly higher than those of TGF-β1 high expression- ELF high expression group (81.3%, 43.8% and 37.5%, *P* = 0.001) and TGF-β1 high expression- ELF low expression group (67.6%, 20.6% and 11.8%, *P* < 0.001) (Figure 4A and B).

Furthermore, we found that the 1-, 3- and 5-year DFS rates of TGF-β1 high expression-ELF low expression (26.5%, 2.9% and 2.9%) were remarkably lower than TGF-β1 high expression- ELF high expression (56.3%, 31.3% and 31.3%, P = 0.002) and TGF-β1 low expression-ELF low expression (60.0%, 60.0% and 37.5%, *P* = 0.002). Also, the 1-, 3- and 5-year OS rates of TGF-β1 high expression-ELF low expression (67.6%, 20.6% and 11.8%) were markedly lower than TGF-β1 low expression-ELF low expression group (90.0%, 70.0% and 60.0%, *P* = 0.003). However, there was no significant difference of OS rates between TGF-β1 high expression-ELF low expression and TGF-β1 high expression- ELF high expression (67.6%, 20.6% and 11.8% vs 81.3%, 43.8% and 37.5%, respectively, *P* = 0.058). We also found no significant difference of DFS and OS rates between TGF-β1 low expression-ELF high expression group and TGF-β1 low expression- ELF low expression group, or between TGF-β1 low expression-ELF low expression group and TGF-β1 high expression-ELF high expression group (Figure 4A and B). Collecting, the results indicated that the combination of TGF-β1 elevation and ELF reduction in HCC tissues appears to be predictive of the poorest prognosis.

## Discussion

In the past few decades, great efforts have been made to explore the molecular mechanism of HCC to identify biomarkers for prediction and to develop effective treatments. In this study, we focused on investigating the prognostic significance of TGF-β1 and ELF, in particular their combination, for HCC. Our first finding showed that the TGF-β1 protein was upregulated in human HCC tissues and no normal liver tissues with strong cytoplasmic TGF-β1 protein immunostaining. The results were consistent with our previous study that the low-expression of TGF-β1 in normal rat liver tissues and the high-expression of TGF-β1 in rat HCC tissues [16]. Like others reports [31,32]. We also found the positive correlation between TGF-β1 and several clinicopathological characteristics: tumor size, tumor number, TNM stage and recurrence. A shorter post-operative survival of HCC patients with high level of TGF-β1 had been documented in this study. The 1-, 3- and 5-year DFS rates and OS rates of HCC patients with high level of TGF-β1 were markedly lower than the low-expression group.

Why do the functions of TGF-β switch from tumor suppression to tumor promotion? Mishra L *et al*. indicated that proper control of TGF-β signaling tumor suppressor function requires an additional adaptor protein, ELF. Research from that group indicated that disruption of ELF expression results in miscolocalization of Smad3 and Smad4, then disruption of TGF-β signaling, allowing normal cells to escape from the regulation of proliferation in carcinogenesis [21,33-36]. However, it was not reported if ELF expression level correlated with survival of HCC patients.

It is therefore of interest to investigate the expression and clinical significance of ELF in patients with HCC. We found that ELF was lost or underexpressed in the majority of HCC tissues, and that a high level of ELF expression predicted a favorable DFS rate and OS rate for HCC patients. Our data showed that the expression of ELF negatively correlated with HbsAg, tumor size, tumor number, TNM and recurrence. The 1-, 3- and 5-year DFS rates of HCC patients with the high level of ELF expression were remarkably higher than those of HCC patinets with the low levels. Similarly, the 1-, 3- and 5-year OS rates of HCC patients with the high level of ELF expression were significantly higher than those of HCC patients with the low levels. These data were consistent with previous studies, which showed that significant ELF reduction was found in HCC, gastric cancer and lung cancer [33-36].

Further, we studied the correlation between ELF and TGF-β1 in HCC patients, and demonstrated their significant negative correlation. Then we used univariate analysis and the Cox regression mode to study the role of ELF and TGF-β1 on HCC, finding that the expression of ELF and TGF-β1 were both significant and independent prognostic factors for DFS or OS of HCC. These data further verified that ELF and TGF-β1 were important and promising candidate tumor biomarker for predicting the prognosis of patients with HCC, and we hypothesized if combination of ELF and TGF-β1 could give us a more sensitive way to predict HCC patients’ outcome.

It is widely understood that a combination of multiple markers might yield more information for predicting clinical outcome of HCC patients [37]. Elevation of TGF-β1 or reduction of ELF in HCC tissues appears to be predictive of a poor prognosis. The combination of TGF-β1 and ELF expression were therefore used as a predictor of clinical outcome. The results indicated that their combination has a better prognostic value compared with either one alone. For example, those patients with low ELF expression and high TGF-β1 expression had the poorest OS and DFS rates, whereas those patients with high ELF expression and low TGF-β1 expression had the most favorable OS and DFS rates. The second best prognosis belonged to these patients with low ELF expression and low TGF-β1 expression. In addition, we found that high level of ELF could partially rescue TGF-β1 related tumor promotion, but TGF-β1still was the more important factor for prognosis of patient with HCC.

## Conclusions

Our study determined that loss or reduction of ELF and elevation of TGF-β1 was correlated with disease progression and metastasis in patients with HCC. And the most interesting finding was that the predictive range of ELF levels combined with TGF-β1 expression was more sensitive than that of either ELF or TGF-β1 alone with regard to OS and cumulative disease recurrence in patients with HCC. From a diagnostic viewpoint, our results suggest that the detection of tumor ELF alone or the combined evaluation of ELF/ TGF-β1 levels could be used as a new prognostic marker in patients with HCC. However, the exact mechanisms of ELF and TGF-β1 expression regulation and function in HCC should been elucidated further. In the future, ELF might be used as potentially powerful target for treatment of HCC through enhancing the tumor suppression of TGF-β pathway.

## Abbreviations

HCC: Hepatocellular cancer
TGF-β: The transforming growth factor β
SBE: Smad-binding elements
ELF: Embryonic Liver Fodrin
β2SP: β2-spectrin
TNM: Tumor-node-metastasis
IRS: Immunoreactivity score
CT: Computed tomography
MRI: Magnetic resonance imaging
PET-CT: Positron emission tomography -CT
DFS: Disease Free Survival
OS: Overall Survival
AFP: Alpha-fetoprotein
HBsAg: Hepatitis B surface antigen
PLT: Platelet
PVTT: Portal vein tumor thrombi. HR, hazard ratio
CI: Confidence interval
